# The New York Institute of Stomatology

**Published:** 1896-06

**Authors:** 

**Affiliations:** The New York Institute of Stomatology


### THE NEW YORK INSTITUTE OF STOMATOLOGY.

A REGULAR meeting of the Institute was held Tuesday evening, April 7, 1896, at the residence of Dr. J. Morgan Howe, 58 West Forty-seventh Street. The President, Dr. Benjamin Lord, in the chair.

The minutes of the last regular meeting were read and approved.

*The President.*—It was thought by the Executive Committee that it would not be wise to call upon the regular committees for reports this evening. We hope they will not be discouraged, because it must be felt by all that it is through these committees that this Institute is to accomplish its best work. We hope the time is not far distant when the committees will be called upon at regular intervals, and their reports thoroughly discussed and criticised.

### COMMUNICATIONS ON THEORY AND PRACTICE.

*Dr. W. St. George Elliott.*—About two months ago one of the surgeons of St. Mary's Hospital came to see me professionally, and incidentally spoke of a boy under his care who had ankylosis of the lower jaw. He asked me to see the case, which I did; I was there during the operation, and it was most interesting. There was complete ankylosis on both sides. The boy was about ten years of age, a Southerner, but there was no history to be obtained of the case. There was imperfect development of the lower jaw, the symphysis being at least an inch back of its normal position. The operation consisted in forming a flap and going into and breaking up the adhesions of the joint, and with mallet and chisel cutting away the end of the ramus and entirely relieving it from its bony attachment. The result of the operation was rather favorable. I took an impression of the upper and lower jaws as far as possible. The mouth was fixed in a semi-open position, probably not quite half an inch, and through that small aperture I introduced a piece of board, on which was some impression compound, and succeeded in getting an impression of the upper and lower jaws. The lower jaw was so short that the lower incisors presented themselves almost horizonally. The general health of the patient was good and he made a very rapid recovery. The one object I had in seeing the case was for the purpose of making a spring splint to give the jaw a certain amount of motion so that it would not become reattached. This was found unnecessary, as the boy voluntarily moved his jaw a good deal. The jaw was forced open an inch or an inch and a quarter. From that time up to the present he has continued to have fair power of mastication. The temporal muscle has been largely lost, but the other muscles continue in good form.

*The President.*—If there are no further communications on theory and practice, we will listen to the paper of the evening, and I have the great pleasure of introducing Professor A. A. Breneman, late Professor of Industrial Chemistry in Cornell University, who will address us on some of the physical properties of the metals, and as all dentists are supposed to be metallurgists—more or less—we may consider it a great favor to have our attention called to the subject by one who has given it special study.

(For Professor Breneman's paper, see page 355.)

*The President.*—I am sure all will agree that Professor Breneman has given us much general information on the properties of metals, and the subject is now before the meeting for discussion, inquiry, and criticism.

*Dr. Elliott.*—I would like to ask Professor Breneman whether it is possible to make an alloy that is perfectly homogeneous, one in which the relative component parts are the same and remain the same in all parts of the ingot.

*Professor Breneman.*—That is an interesting question, and it touches the phenomenon of *liquation* or the separation of the elements of the alloy during cooling. The matter of preserving perfect uniformity in the mass of some alloys, however carefully and thoroughly they have been made, so that they shall remain exactly the same after cooling, is a difficult one. There is a tendency in the more fusible elements in a liquid mass to separate themselves as the less fusible portions become hard, and so to gather in the centre of the mass, the part last to cool, and there to form a mixture or compound of different composition from the portions first to cool. This has been the bugbear of metal-workers in many different lines. The prevention of it depends in special cases upon special devices; no general method can be given. If the cooling mass within its mould could be continuously shaken during its cooling we should probably have the best approach to uniformity in the mass, but this is generally impracticable. Frequently a slight change in chemical composition will reduce the tendency to separation. Some processes of working metals are based not upon preventing, but upon encouraging this tendency. The tendency of the ingredients to separate during the hardening of a liquid mass is common to all forms of matter. A familiar case is that of water in freezing. It has long been known that the impurities in a freezing mass of water are pushed together so as to concentrate in the portion last frozen. This was a serious difficulty in the early operation of artificial ice-plants. If the water was not thoroughly purified the ice at the centre of the block, or the portion last frozen, would contain impurities sufficient to injure the entire mass of ice. The remedy for this, in the case of artificial ice-making, is, of course, to have the water as pure as possible. The water should be distilled, and distilled under conditions which will regulate its purity. To return again to what happens in a cooling mass like this, I should say that as the molecules build themselves up into a solid state, often into a crystalline state, the more fusible portions, which will also be the more mobile under those conditions, may be crowded out, as it were, and escape through orifices left in the hardening mass.

*Dr. E. A. Bogue.*—Is there any way by which metals so widely different in the melting-point as silver, tin, and zinc may be alloyed and made homogeneous?

*Professor Breneman.*—The question is somewhat complicated by bringing in three metals. There is no inherent difficulty brought in merely by wide differences of melting-point. Metals that differ most widely in their fusing points may very readily alloy. The difficulty when it occurs is one depending rather upon an unknown physical peculiarity of the metal, and if it cannot be conquered by the use of another metal which serves as a mediator between the two it is difficult to conquer. Certain metals in very minute proportions will effect a kindly union of metals otherwise apparently incompatible. In certain cases this may be merely a reducing action, where the mediating metal serves to reduce portions that exist as oxides and keep the metals apart, keeping their surfaces from uniting. This, I think, is the most general explanation of this mediating action, but it is an entirely general explanation. The day may come when tables may be made in which, with the proportions of the elements varying uniformly on a scale of equal parts, we may find without experiment the properties to be expected from mixing given proportions of metals.

*Dr. S. E. Davenport.*—I should like to ask Professor Breneman whether there is a scientific foundation for what I seem to have observed within the past few weeks, or whether I have allowed my imagination to run riot. During my professional life I have been using the so-called redistilled mercury supplied in the various dental depots for amalgamated alloys. Recently it has seemed to me a matter of sufficient importance to make an effort to get the mercury purified to a greater degree, and I have succeeded in interesting a chemist in the question. He has supplied me with mercury as pure, he says, as it is possible to make it, using some peculiar processes which, no doubt, Professor Breneman knows all about. As I have been using the same alloy, for most of my amalgam fillings for more than ten years, it seems to me that I ought to be able to recognize even slight changes. With this purified mercury the color of the filling is improved, less mercury is needed to incorporate the filling, and the mixed mass seems more homogeneous and finely granular.

*Professor Breneman.*—Perfectly clean mercury is very difficult to obtain; primarily because of metallic impurities which, while they are mainly separated by distillation, are not so easy to separate absolutely; and minute traces of foreign metals may greatly affect the qualities of the mercury. Perfectly clean mercury is necessary in many physical experiments. When we confine a mass of mercury and measure its volume in a graduated scale we want the mercury to make a sharp meniscus, as it is called,—that is, to curve upward. If it contains impurities, or if the tube is dirty, the mercury instead of making a clean upward curve will be almost flat in its surface within the tube. To purify mercury we resort, first of all, to distillation and redistillation; but sometimes we find that a small quantity of impurity will persist even after distillation. There is a peculiar power existing in certain liquids of carrying over as they boil the vapors of other liquids which in themselves have a much higher boiling-point; a great deal of one vapor will carry over a little of the other, and that little may be a harmful quantity. There is also greasy matter, probably some hydrocarbon-oil which has a boiling-point near that of mercury, therefore very difficult to separate by distillation; and such impurities may very much impair the value of mercury in use. Where distillation does not answer, chemical purification may be resorted to. The action of dilute nitric acid, while it will dissolve a very little of the mercury, will dissolve all of the contaminating metals. Other oxidating solutions, like sulphuric acid and bichromate mixture, will serve. Sometimes there are minute mechanical impurities which can be gotten rid of by filtration through cotton under pressure, or through a thin layer of porous wood under high pressure. Generally chemists find one or the other of these means to answer the purpose, but sometimes many means have to be adopted, and occasionally it is extremely difficult to get thoroughly clean mercury.

*The President.*—Will Professor Brĕneman tell us whether he thinks aluminum will come into use in the arts or in manufactures to any considerable extent?

*Professor Breneman.*—I think that until we have a cheaper method of manufacturing aluminum it cannot possibly displace the use of iron to any great extent, although there are many cases in which its lightness and its beauty, and its freedom from attack, are elements worth paying for. The production of electricity—and aluminum is manufactured by a process of electrolysis—is at best an expensive process. Even when power in such quantities as is supplied at Niagara Falls is to be had it is not procured for nothing. The great tunnel, the immense power-houses, the tremendous dynamos set up at Niagara for the making of aluminum and other things cost a great deal of money and the wear and tear is great. Besides that, the ores from which aluminum is made at present are not very abundant nor very cheap. While common clay contains aluminum in great quantities, there is at present no practical method of separating it from the clay. However cheap the raw material may become, it is unlikely that any process of working it by electricity, a process which uses carbon poles, themselves rapidly destroyed, which uses an agent requiring expensive machinery, and which makes its products by the single ton instead of by hundreds of tons, will ever be a dangerous competitor with iron manufacture. Aluminum to-day costs, by the cheapest process, about fifty cents a pound. It will be seen that the interval which separates it as a competitor from iron at a cent and a quarter a pound or less is very great.

A vote of thanks to Professor Breneman for his very interesting and instructive address was given.

Adjourned.

S. E. Davenport, D.D.S., M.D.S.,
*Editor The New York Institute of Stomatology.*

